# Optimum Design of a Composite Optical Receiver by Taguchi and Fuzzy Logic Methods

**DOI:** 10.3390/mi12121434

**Published:** 2021-11-23

**Authors:** Ning Wang, Xing Peng, Lingbao Kong

**Affiliations:** 1 Department of Ophthalmology, Shanghai General Hospital, School of Medicine, Shanghai Jiaotong University, Shanghai 200080, China; drwangning@126.com; 2 Shanghai Engineering Research Center of Ultra-Precision Optical Manufacturing, School of Information Science and Technology, Fudan University, Shanghai 200433, China; pengxing@fudan.edu.cn

**Keywords:** LED, visible light communication, optical receiver, Taguchi method, fuzzy logic, field of view

## Abstract

This paper investigates a composite optical receiver for an indoor visible light communication (VLC) system. The optical gain, received power, and signal-to-noise ratio (SNR) are considered to be optimized. However, it is difficult to find a balance between them in general design and optimization. We propose the Taguchi and fuzzy logic combination method to improve multiple performance characteristics effectively in the optical receiver. The simulated results indicate that the designed receiver has the characteristics of an optical gain of 10.57, a half field of view (HFOV) of 45°, a received power of 6.4635 dBm, a signal-to-noise ratio (SNR) of 89.8874 dB, and a spot size of 2 mm. The appropriate weights of the three performance characteristics for the inputs of the fuzzy controllers increase the optical gain by 13.601 dB, and the received power and SNR by 11.097 dB and 0.373 dB, respectively. Therefore, the optical receiver optimally designed by the Taguchi and fuzzy logic methods can significantly meet the requirements of an indoor VLC system.

## 1. Introduction

In the 1980s, solid-state lighting for illuminance was gradually replaced with the development of high-efficiency yellow, red, and orange light-emitting diodes (LEDs) [1,2]. As LED lights have some obvious advantages in their low carbon emissions, contain no mercury, and are power efficient and durable, they have been widely utilized in medical applications, indoor farming and plantation, information display, and so on [3,4]. Additionally, the potential function of simultaneously offering illuminance and communication with high response sensitivity and switching frequency can significantly solve the scarcity of radio frequency (RF), and provide a reliable communication system [5,6,7]. Thus, visible light communication (VLC) technology came into being.

VLC is a new paradigm that can completely change the future of wireless communication. With the characteristics of a high communication rate, unlimited bandwidth, and being free from electromagnetic interference, VLC systems have been applied in intelligent transportation [8], indoor wireless communication [9,10,11], smart cities [10], human sensing [11], vehicular communication [12], and location in robotics/warehouses [13]. VLC systems consist of a transmitter, an optical receiver such as a photodetector or image sensor, and a free-space optical communication channel. Presently, various pieces of research have mainly focused on the channel coding, modulation mode, and layout optimization of the light source, but not on the optical receiving system in VLC [14]. However, a suitable optical receiver designed for the VLC system can significantly improve the receiving energy, optical gain, and signal-to-noise ratio (SNR), and can provide a guarantee for a high communication rate [15,16]. The optical receiver is often utilized to collect and transmit more lights to the photoelectric detector [17], and it requires high optical gain and a wide FOV. In most cases, the receivers only consist of a filter and a cover lens, and the communication quality cannot be guaranteed. Some traditional optical receivers, such as compound parabolic concentrator (CPC), have a tradeoff between FOV and optical gain [18,19,20]. The Cassegrain antenna has quite a small FOV and cannot meet the requirements of the VLC systems [21]. The Fresnel lens has a strict limitation on the incidence angle [22,23]. Additionally, some novel optical receiving systems have been proposed, such as the continuous zoom antenna designed for mobile VLC [16], the gradient-index lens with a CPC shape [15], and the planer concentrator for the improvement of FOV and received power [24].

In this regard, we have previously proposed a novel optical receiving antenna based on a compound parabolic concentrator for an indoor VLC system, and analyzed the optical gain, received power, and SNR distribution [22]. Our results show that the designed optical receiver is effective in collecting energy from the Lambert source. However, the spot size is 8 mm and the received power is dispersed over the receiving surface. According to the optical characteristics of the designed receiver, for a fixed FOV, the larger the exit surface, the more energy can be collected. However, due to the small physical size of the detector, the energy focused on the receiver cannot be effectively used in the communication system. Moreover, the previously proposed receiver was optimized by the Taguchi method, which aims to deal with the optimization of single performance characteristics. For the optimization of processes with multiple performance characteristics, the usual suggestions are left to engineering judgment, and are verified by experiments. Thus, it is difficult to find a balance between multiple performance characteristics such as FOV, optical gain, received power, and SNR. Optimizing only one certain optical characteristic may not meet the image quality requirements, which hinders the improvement of the optical performances of the receiver and the communication stability of the VLC system. The definition of performance characteristics, such as the lower the better or the higher the better, contain a certain degree of uncertainty and ambiguity. Thus, besides proposing novel design ideas, it is necessary to optimize multiple quality characteristics with the appropriate method for an optical receiver.

Therefore, we propose and demonstrate a novel composite optical receiver with the Taguchi and fuzzy logic combination method. The proposed novel optical receiver is composed of two stages: the lens-walled CPC and the hemispherical lens (HL). The effects of varying the bottom wall height H, rotation degree β, spacing distance L between the two stages, and hemispherical radius R are studied to improve the characteristics of the receiver. By utilizing the Taguchi and fuzzy logic methods, the optimization of complex multi-performance characteristics can be transformed into the optimization of single multiple performance characteristics indices (MPCI), which have the potential to efficiently and reliably obtain a wide FOV, high optical gain, well-received power, and SNR. The results indicate that the optimized receiver has a wider FOV, higher gain, and ensures better received power and SNR than the initial structure by utilizing the proposed optimization method. Furthermore, the optical efficiency and received spot area have been improved simultaneously. The design scheme of the composite optical receiver is briefly described in Section 2. Section 3 proposes the Taguchi and fuzzy logic methods applied to the receiver optimization and evaluation. Section 4 presents the channel performance evaluation and comparison. Finally, the conclusions are illustrated in Section 5.

## 2. Design of the Composite Optical Receiver

The traditional mirror CPC is a non-imaging concentrator designed with the principle of edge rays [25]. In 1974, Prof. Winston invented the CPC, which consists of two symmetrical paraboloids rotating around a symmetry axis. Until now, many kinds of research have been about the application of CPC in VLC systems [26]. However, the research indicates that the tradeoff between the optical gain and FOV of CPC limits the optical performance. Therefore, several novel optical receivers based on gradient-index lenses [15], continuous zoom lenses [16], and CPC structures [18] have been proposed, which can effectively improve the FOV and guarantee a high optical gain, ensuring better received power and SNR. However, the light spots obtained by the proposed structures are relatively scattered, and the optical energy distribution is not concentrated enough, while the photodetector size is quite small in VLC systems. Increasing the concentration ratio and reducing the spot area is significant to improve the utilization of optical energy and ensure the stability of the communication. However, there have been some limitations on obtaining the best structure combinations of receivers effectively with multiple quality characteristics. Meanwhile, in the field of optical receiver design for VLC systems, there is hardly any research regarding receiver optimization or optimization methods, while an appropriate optimization methodology can significantly improve the efficiency of the receiver design and obtain better communication performance in VLC systems. Therefore, we further propose the composite optical receiver, and mainly research the optimization method based on Taguchi and fuzzy logic.

**Figure 1:**
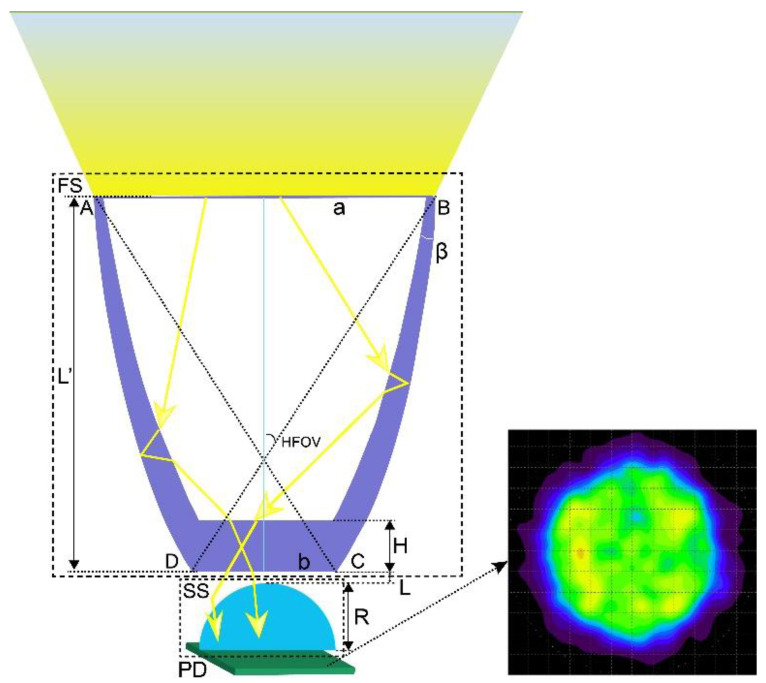
Schematic diagram of the proposed composite optical receiver. FS: first stage of the receiver; SS: second stage of the receiver; AB: incident plane; DC: exit facet of the FS; a: inlet radius; b: outlet radius; β: rotation degree of the FS; H: bottom wall’s height of the FS; L: spacing distance between the FS and SS; R: hemispherical radius of the SS.

Figure 1 presents the proposed optical receiver for indoor VLC systems, combining the best of both the lens-walled CPC in [22] and the hemispherical lens (HL). The rays track through the lens-walled CPC with reflection and refraction, which is incident on the surface of the HL. The rays can be focused into a small area, which significantly improves the optical energy receiving efficiency. Furthermore, the proposed optical receiver consists of four parameters: the bottom wall’s height H, rotation degree β, spacing distance L, and hemispherical radius R, which can be optimized by Taguchi and fuzzy logic methods to obtain the best structural parameter combination. The initial structure of the receiver in this study has the following parameters: a β of 3°, H of 4.0 mm, L of 2.0 mm, and R of 3.5 mm, and all these parameters need further optimization.

Parameters a and b are the radius of the inlet and outlet of the CPC, respectively; the concentration *Cg* can be expressed as [18]:

(1) $$C_{g}\;=\;\frac{a}{b}\;=\;\frac{1}{sin\theta_{max}}$$

where *θ_max_* represents the HFOV of the CPC, and any ray with an incident angle smaller than the half acceptance angle *θ_max_* can reach the outlet aperture of the CPC.

The focal length of the parabolae AC and BD can be calculated by Equation (2):

(2) $$f\;=\;b\left(1\;+\;sin\theta_{max}\right)$$

Meanwhile, according to the calculated *f* and HFOV, the height of the CPC can be expressed by Equation (3) as:

(3) $$L\;=\;\frac{b(1\;+\;\sin\theta \max)\cos\theta max}{\sin^{2}\theta max}\;=\;\frac{f\cos\theta max}{\sin^{2}\theta max}$$

The CPC with a length of *L* is called the standard CPC. However, in practical applications, the CPC is often truncated; that is, the upper part of the CPC is cut off and the length is reduced. The interception ratio of the CPC can be expressed as *k*:

(4) $$k\;=\;\frac{L^{\prime }}{L}$$

where *L’* is the height of the CPC after truncation, and appropriate truncation has little influence on the performance of the CPC. The lens-walled CPC is formed by rotating a symmetrical mirror CPC around the top end points A and B, respectively, toward the inside by a certain rotation degree β. The area between the original CPC curves and the new CPC curves is the lens, which is filled with the material of PMMA. The outside surface of the lens is layered with a reflective coating, which allows rays to experience both reflection and refraction.

**Figure 2:**
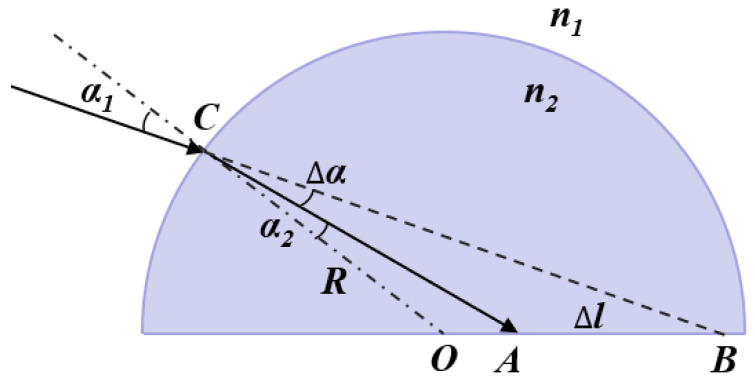
Diagram of the HL.

Shown in Table 1 are the basic parameters of the CPC structure applied in this paper. We set the HFOV to be 20°. The HL is a widely-used non-imaging concentrator with omnidirectional gain and a wide FOV. Figure 2 presents the schematic diagram of the HL. The refractive index of the HL is n_2_ and the medium index is n_1_. The radius of the HL is represented as R. Assuming a light incident at point C with an incident angle α_1_ on the HL, it is refracted to point A with a refractive angle of α_2_. The relationship between α1 and α_2_ is represented as follows [17]:

(5) $$\frac{\sin\alpha_{1}}{\sin\alpha_{2}}\;=\;\frac{n_{2}}{n_{1}}$$

The deflection angle is

(6) $$∆\alpha \;=\;\alpha_{1}\;-\;\alpha_{2}\;=\;\alpha_{1}\;-\;sin^{-1}(n_{1}\sin\frac{\alpha_{1}}{n_{2}})$$

Upon applying the sine theorem to triangles CBO and CAO, the relationship between the different angles and sides can be expressed as follows:

(7) $$\left\{\begin{array}{c} \frac{\sin\alpha_{1}}{\bar{𝑂B}}\;=\;\frac{\sin∠\mathrm{CBO}}{R}\\ \frac{\sin\alpha_{2}}{\bar{𝑂A}}\;=\;\frac{\sin∠\mathrm{CAO}}{R} \end{array}\right.$$

where $∠\mathrm{CBO}\;=\;\alpha_{1}\;-\;\alpha_{2}\;+\;∠\mathrm{CBO}$.

Therefore, the length of $\bar{𝑂A}$ can be calculated as

(8) $$\bar{𝑂A}\;=\;\frac{R\sin\alpha_{2}}{\sin\left(\alpha_{1}-\alpha_{2}+∠\mathrm{COB}\right)}$$

Furthermore, the deflection distance of the incident light in the HL can be expressed as follows:

(9) $$∆l\;=\;\bar{𝑂B}\;-\;\frac{R\;n_{1\;}\sin\alpha_{1}}{n_{2}\;\left(\alpha_{1}\;-\;sin^{-1}\left(n_{1}\sin\frac{\alpha_{1}}{n_{2}}\right)\;+\;sin^{-1}(\frac{R\sin\alpha_{1}}{\bar{𝑂B}})\right)}$$

As illustrated in Equation (9), if a light ray is incident on the HL with a certain angle α1, then the deflection distance ∆l mainly depends on the R, $n_{1}$ and $n_{2}$, which significantly reduce the spot area by choosing suitable materials of the HL.

## 3. Optimization of the Composite Optical Receiver

**Figure 3:**
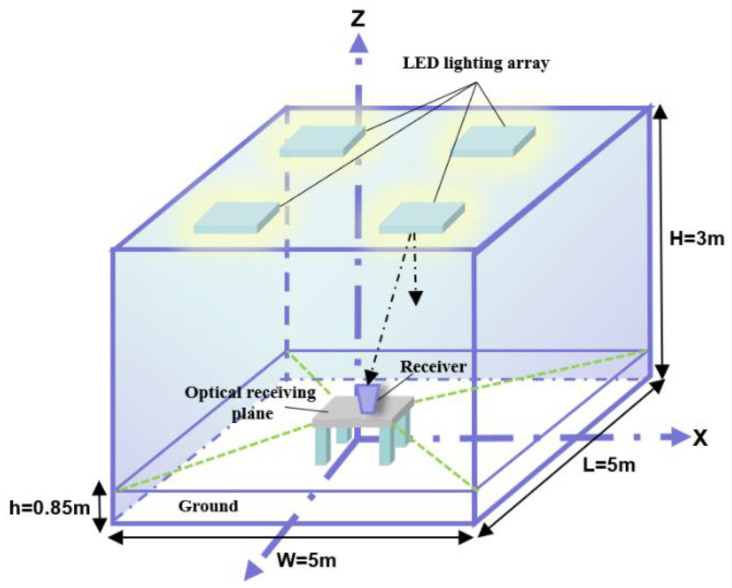
The communication model in the VLC systems.

There are four parameters of the composite optical receiver which significantly affect the performance characteristics, as discussed in Section 2. The software of Tracepro and Matlab were utilized to perform the optimization and evaluation of the proposed composite optical receiver in indoor VLC systems. The simulation was operated in a 5 m × 5 m × 3 m room with four 60 × 60 LEDs array units, as shown in Figure 3. The LED’s optical power is 20 mW, the center intensity is 0.73 cd, and the LED unit spacing is 10 mm. The LED array was placed by the principle of minimum mean square error of illumination, and the center coordinates of the four LEDs array units were (0.815, 0.815), (4.185, 0.815), (4.185, 4.185), and (0.815, 4.185), respectively.

Four structural parameters have effects on the optical receiver, which means many experimentations need to be explored. Meanwhile, multiple performance characteristics cannot simultaneously achieve the optimal solution. However, the Taguchi method can effectively obtain a large amount of experimental information with the least number of experiments, and can interpret experimental results faster than traditional methods. Furthermore, fuzzy logic control can be utilized to model uncertain and complicated issues. Fuzzy logic can effectively define the relationship between the system input and the expected output, and can convert the evaluation result into a total evaluation coefficient of MPCI. Therefore, we combine the advantages of Taguchi experiments and the fuzzy logic method to optimize and evaluate the designed optical receiver.

### 3.1. Taguchi Experimentation

The target of the Taguchi experiment is the achievement of optimum performances by applying suitable parameter combinations, utilizing an orthogonal array to analyze the results of different combinations [27]. The Taguchi method has the advantages of reducing production time and costs by utilizing the orthogonal array [28]. The most influential parameters are arranged in an orthogonal array, thus greatly reducing the number of experiments. Meanwhile, these parameters are commonly referred to as “control factors”, and each of these parameters can have a specified number of design settings, called “level settings” [27,28,29]. As shown in Table 2, in the Taguchi experiments, the quality characteristics of the optical receiver are determined by four factors, namely: (A) the rotation angle β; (B) the bottom wall’s height H; (C) the spacing distance L; (D) the hemispherical radius R. Each of these four factors is assigned three possible level settings.

The objective quality characteristics include optical gain G, the received power P, and the SNR of the optical receiver. As shown in Table 3, the Taguchi experiment is configured in the L9 (3^4^) orthogonal array. Meanwhile, in Taguchi’s method, a loss function is defined to calculate the simulated noise intensity, which represents the deviation between the experimental value and the expected value, and its unit is the decibel (dB). Regardless of the characteristics of the serial number, the larger the serial number, the smaller the change and the higher the quality. Additionally, the S/N ratio is summarized as “the larger the better” or “the smaller the better”, which is expressed as follows [30]:

(10) $$\mathrm{Larger}\;\mathrm{the}\;\mathrm{better}:\;\eta \;=\;-10log\frac{\sum_{i=1}^{n}\frac{1}{y_{i}{}^{2}}}{n}$$

(11) $$\mathrm{Smaller}\;\mathrm{the}\;\mathrm{better}:\;\eta \;=\;-10log\frac{\sum_{i=1}^{n}y_{i}{}^{2}}{n}$$

where $y_{i}$ represents the values of the G, P, or SNR related to the *i*-th trial, and n represents the total number of Taguchi experiments.

**Figure 4:**
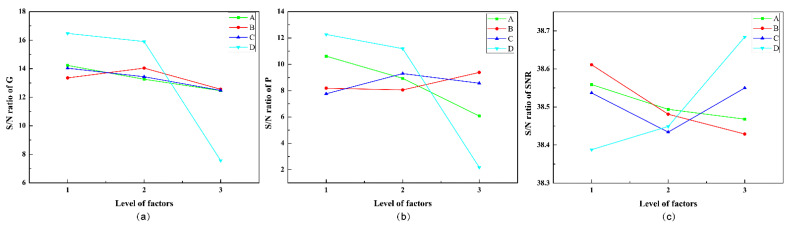
S/N ratio of the different characteristics. (**a**) S/N ratio of the G at different levels; (**b**) S/N ratio of the P at different levels; (**c**) S/N ratio of the SNR at different levels.

**Figure 5:**
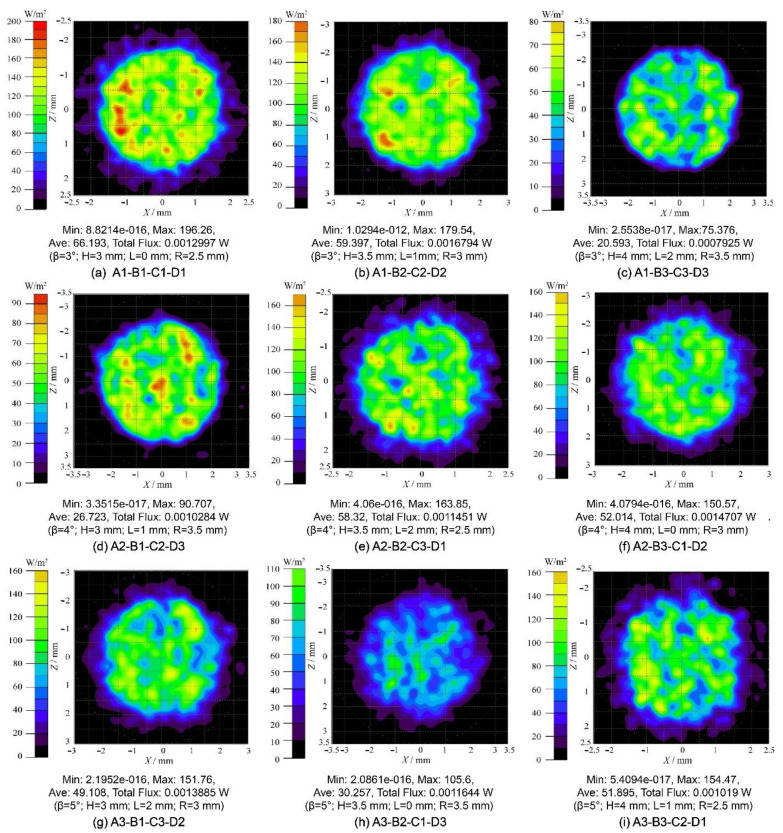
Diagram of the received irradiance distribution for the different parameter combinations of the optical receiver.

**Figure 6:**
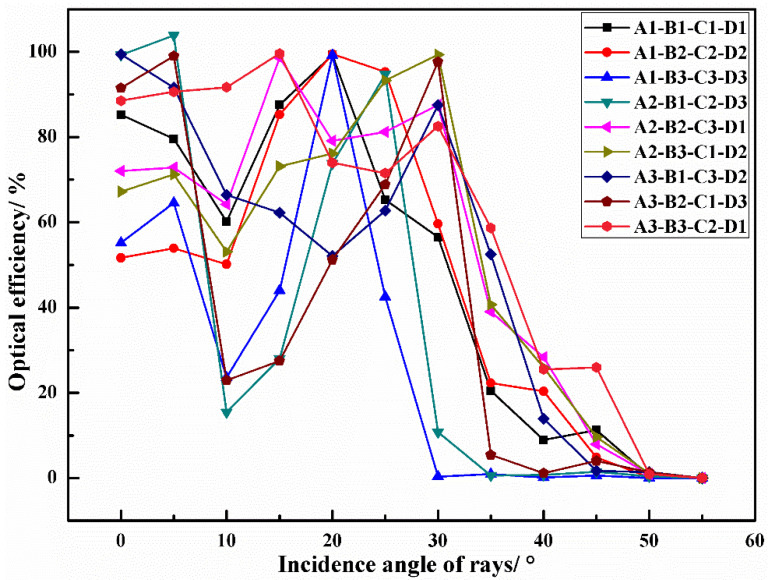
The relationship between the light efficiency and the incident angle under different structural settings of the optical receiver.

Consequently, the larger-the-better S/N ratio is utilized to evaluate the control factorial combinations in the increase of the G, P, and SNR for the optical receiver. In this paper, A1 means control factor A (Rotation degree β) and the level setting “1” (β = 3°), and so do other combinations of letters and numbers. As presented in Figure 4, the S/N ratio at different levels varies with different control factor combinations. By identifying the maximum S/N ratio, the optimal structural parameters can be obtained conveniently. Therefore, the most effective parameter combination for G optimization is: A1 (β = 3°), B2 (H= 3.5 mm), C1 (L = 0), and D1 (R = 2.5 mm). The experiment was verified by the parameter combination A1-B2-C1-D1, and it was found that G is 8.62, which is significantly higher than other combinations shown in Table 3. Thus, from the point of view of G, the parameter combination A1-B2-C1-D1 stands for the optimum structure. Similarly, the optimal structural parameter combination of optimization P is determined by: A1 (β = 3°), B3 (H = 4.0 mm), C2 (L = 1.0 mm), and D1 (R = 2.5 mm). The *p*-value of the structure settings A1-B3-C2-D1 is 5.0730 dBm, which is higher than the *p*-value of other original Taguchi experiments. However, under these structural parameters, G is just 6.84. In addition, the most effective structural parameters for the optimization of the SNR are: A1 (β = 3°), B1 (H = 3.0 mm), C3 (L = 2.0 mm), and D3 (R = 3.5 mm). The SNR value of this structure combination is 86.4925 dB, while the G is 7.94, and the P is 4.3285 dBm. The structure settings A1-B1-C3-D3 have great performance on the SNR, whereas the G and P cannot maintain a high level simultaneously. The received irradiance distribution of the optical receiver is presented in Figure 5. In the Taguchi experiment, the optical receiving energy and spot area vary with different parameter combinations. The different sizes and shapes of the spots indicate that the receptacles set by different structures have different abilities to concentrate light energy. The structural settings A1-B1-C1-D1 and A1-B2-C2-D2 both have smaller spot sizes and greater optical energy. Figure 6 shows the optical efficiency with different combinations of structural parameters in the Taguchi experiment. The optical efficiency with different experimental settings has similar trends. However, there are also significant differences between the nine experimental settings for the FOV attribute. In the case of A3-B3-C2-D1, the optical efficiencies are 58.66%, 25.51% and 25.97% when the incident angles are 35°, 40° and 45°, respectively. In the case of A3-B1-C3-D2, the optical efficiency is 52.46%, 13.93% and 1.70% respectively, whereas in A1-B3-C3-D3, the optical efficiency with these three incident angles is close to 0. Different Taguchi experimental combinations have different optical efficiency performances; the designed optical receiver can significantly improve the FOV. In some structural settings, even if the incident angle is large enough, the optical efficiency can still be maintained up to 40%.

### 3.2. Fuzzy Logic Method

**Figure 7:**
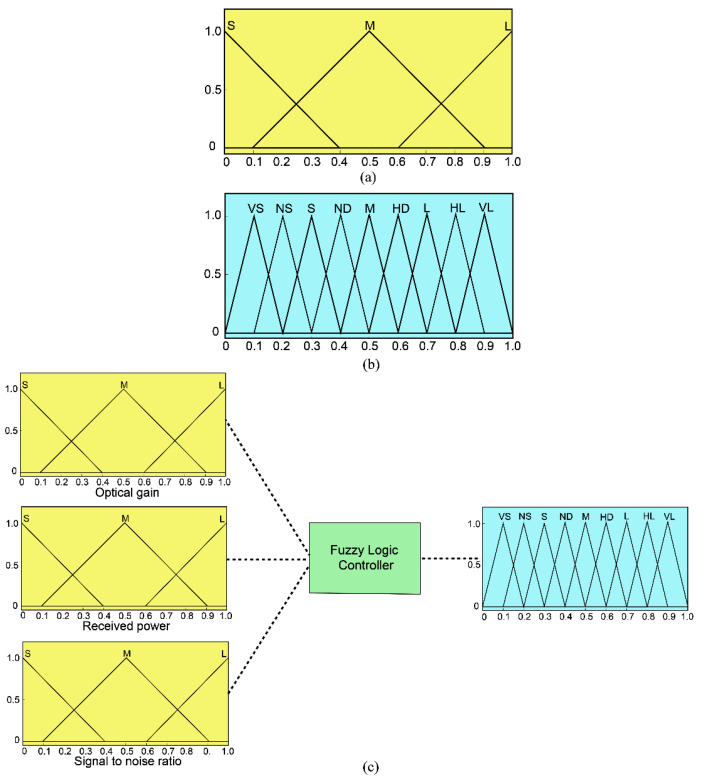
Membership functions for the quality characteristics of the G, P, and SNR (**a**); membership functions for the multi-response performance index (**b**); structure of the fuzzy logic unit (**c**).

Fuzzy logic is a mathematical theory of imprecise reasoning. It uses language terms to simulate the human reasoning process, and is appropriate for the definition of the connection system’s input and output [31,32]. Fuzzy controllers and fuzzy reasoning can deal with uncertainty, and have been widely used in some complex industrial systems [33]. The fuzzy logic system mainly consists of an inference engine containing a rule base and a database, a fuzzifier, and a de-fuzzifier [32]. The performance evaluation can be normalized after the Taguchi experiment. Then, the crisp inputs can be converted into fuzzy sets by the fuzzifier with the membership function, and the inference engine can obtain fuzzy values by performing fuzzy reasoning on fuzzy rules. In addition, the fuzzy value is converted into a clear output by utilizing the de-fuzzifier. In general, the membership function can determine the fuzzy value by defining the membership degree of the object, but the standard method cannot choose the appropriate membership function shape for the fuzzy set of control variables. In this paper, the Mamdani implication method is used to make fuzzy inferences on the fuzzy rules. Each fuzzy rule with the statement of “if–then” can be represented as [32]

(12) $$\left\{\begin{array}{c} Rule\;1:If\;Y_{1}\;is\;D_{1}\;and\;Y_{2}\;is\;E_{1\;}and\;Y_{3}\;is\;F_{1},\;\;\;\;\;then\;Z_{1}\;is\;G_{1};\\ \begin{array}{c} Rule\;2:If\;Y_{1}\;is\;D_{2}\;and\;Y_{2}\;is\;E_{2\;}and\;Y_{3}\;is\;F_{2},\;\;\;\;\;then\;Z_{1}\;is\;G_{2};\\ \ldots \end{array}\\ Rule\;n:If\;Y_{1}\;is\;D_{n}\;and\;Y_{2}\;is\;E_{n\;}and\;Y_{3}\;is\;F_{n},\;\;\;\;\;then\;Z_{1}\;is\;G_{n}; \end{array}\right\}$$

where Y_1_, Y_2_, and Y_3_ represent the inputs; Z_1_ is the output; and D_i_, E_i_, and F_i_ represent the fuzzy subsets defined by membership functions μ_Di_, μ_Ei_, and μ_Fi_, respectively. Figure 7a,b shows the membership functions, which use triangular membership functions. The M rule is based on the Mamdani implication reasoning method to reason a set of disjunctive rules, which can be represented as follows:

(13) $$\mu_{G0}\left(Z\right)\;=\;(\mu_{D1}\left(Y_{1}\right)\;\wedge \;\mu_{E1}\left(Y_{2}\right)\;\wedge \;\mu_{F1}\left(Y_{3}\right)\;\wedge \;\mu_{G1}\left(Z\right))\;\vee \;\ldots \;(\mu_{Dn}\left(Y_{1}\right)\;\wedge \;\mu_{En}\left(Y_{2}\right)\;\wedge \;\mu_{Fn}\left(Y_{3}\right)\;\wedge \;\mu_{Gn}\left(Z\right))$$

Figure 7c illustrates the flow chart using fuzzy logic theory. In this study, the fuzzy value is transformed into a clear output value by defuzzification. In addition, the center of gravity method is the most commonly used fuzzy output function defuzzification method in this research. Moreover, the fuzzy inference output $\mu_{G0}\left(Z_{i}\right)$ can be converted to the non-fuzzy value Z_0_, and its expression is as follows:

(14) $$Z_{0}\;=\;\frac{\sum_{i=1}^{k}Z_{i}\mu_{G0}\left(Z_{i}\right)}{\sum_{i=1}^{k}\mu_{G0}\left(Z_{i}\right)}$$

Before fuzzy logic analysis, the initial SNR of the G, P, and SNR obtained in the Taguchi experiment should be normalized between 0 and 1 for the consideration of smaller ranges. The $Y_{i}\left(k\right)$ describes the S/N ratio of the G, P, and SNR, and k denotes the values of G, P, and SNR. The normalization can be represented as follows:

(15) $$\hat{Y_{i}}\left(k\right)\;=\;\frac{Y_{i}\left(k\right)\;-\;min_{k}Y_{i}\left(k\right)}{\left[max_{k}Y_{i}\left(k\right)\right]-\left[min_{k}Y_{i}\left(k\right)\right]}$$

**Figure 8:**
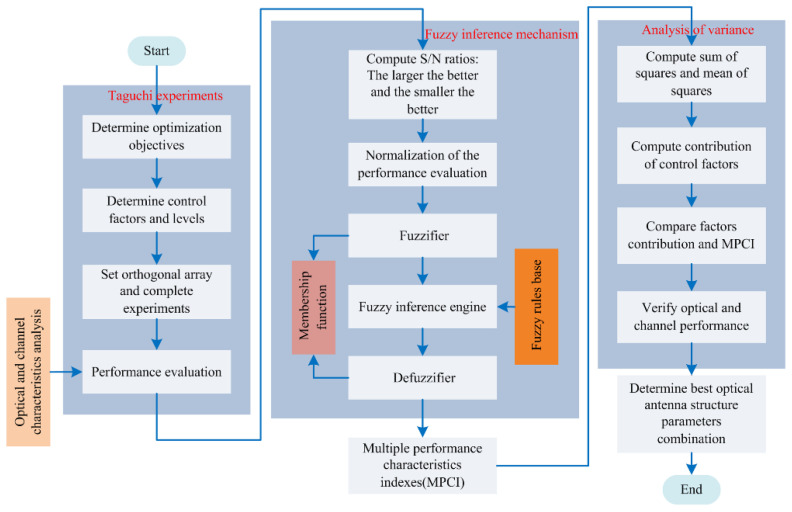
Schematic diagram of the flowchart for the Taguchi and fuzzy logic methods.

Figure 8 presents the schematic diagram of the flowchart for the Taguchi and fuzzy logic methods. The methods proposed to optimize the optical receiver are composed of the following steps:

(a) Choose the orthogonal array L9(3^4^) as the starting point of the Taguchi experiments.
(b) Convert each quality feature to S/N (Table 3), and then normalize (Table 4).
(c) Set the input and output membership functions and fuzzy rules (Table 5).
(d) Start to calculate the fuzzy controller (fuzzy reference, defuzzification interface).
(e) Calculate the values of MPCI (Table 6).
(f) Perform an analysis of variance (ANOVA) on the main component points.
(g) Draw MPCI response tables and charts to determine the best combination of parameters.
(h) Perform confirmation tests.

**Figure 9:**
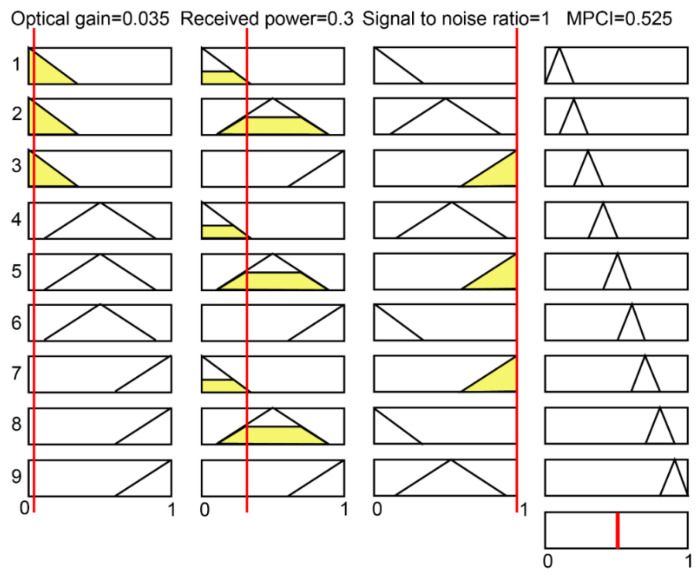
The fuzzy logic reasoning procedures for the optical receiver.

**Figure 10:**
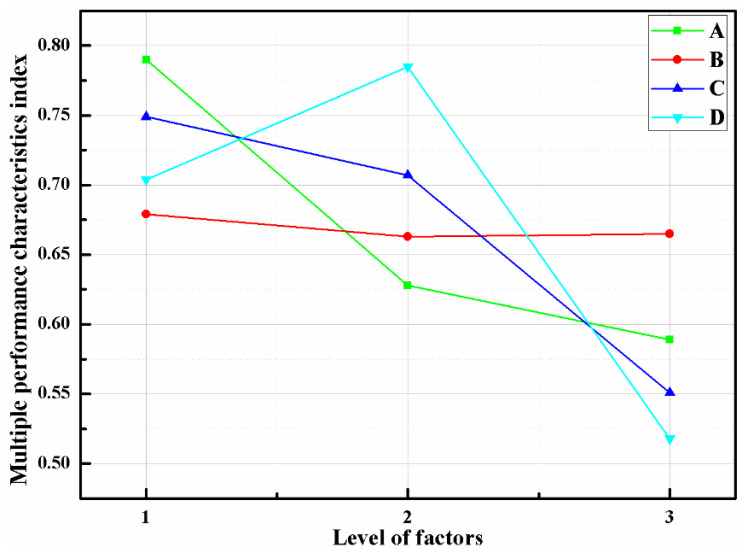
Response graph of the MPCI values with the control factors A (rotation angle β), B (bottom wall height H), C (spacing distance L), and D (hemispherical radius R) at the three level settings.

Table 4 lists the normalized results of G, P, and SNR. In order to optimize the multiple performance characteristics of the optical receiver, the input of the fuzzy logic structure can be integrated as the total evaluation coefficient of the MPCI. As shown in Figure 7c, the G, P, and SNR are the input variables of the fuzzy logic unit for the final total of the MPCI value. There are three fuzzy sets for the inputs S, M, and L, while the outputs have nine sets: VS, NS, S, ND, M, HD, L, HL, and VL. Presented in Table 5 are the fuzzy rules used for the fuzzy logic controller. The MPCI values of the Taguchi experiments are presented in Table 6. For instance, Figure 9 describes the fuzzy logic reasoning procedures for the optical receiver; the normalization values of the G, P, and SNR are 0.035, 0.3, and 0, respectively, and the MPCI value is 0.525. Furthermore, as presented in Table 7, by calculating the average value of control factors A, B, C, and D on MPCI, the influence of them can be obtained conveniently. Figure 10 shows the response graph of the MPCI values with the control factors A (rotation angle β), B (bottom wall height H), C (spacing distance L), and D (hemispherical radius R) at the three-level settings. The results show that the optimal level of each control factor is A1 (β = 3°), B1 (H = 3.0 mm), C1 (L = 0), and D2 (R = 3.0 mm) for the maximum MPCI values. In addition, a larger MPCI value indicates a smaller difference in the performance characteristics. Meanwhile, the relative importance of the β, H, L, and R for the G, P, and SNR must be known, in order to determine the optimal parameter combinations more accurately.

### 3.3. Variance Analysis

Variance analysis can significantly separate the changes caused by experimental errors, and can determine the changes of various control factors [34]. The analysis of variance is similar to the “max–min” analysis shown in Table 7. The variance analysis results are presented in Table 8. Through the analysis of variance, we can see the influence of the control factors on a variety of performance characteristics. Factor D accounted for 39.23% of the total variance of MPCI. Meanwhile, the contribution of factors A, B, and C accounted for 29.46%, 2.34%, and 28.97%, respectively. In addition, the analysis results are similar to the results in Table 7.

**Figure 11:**
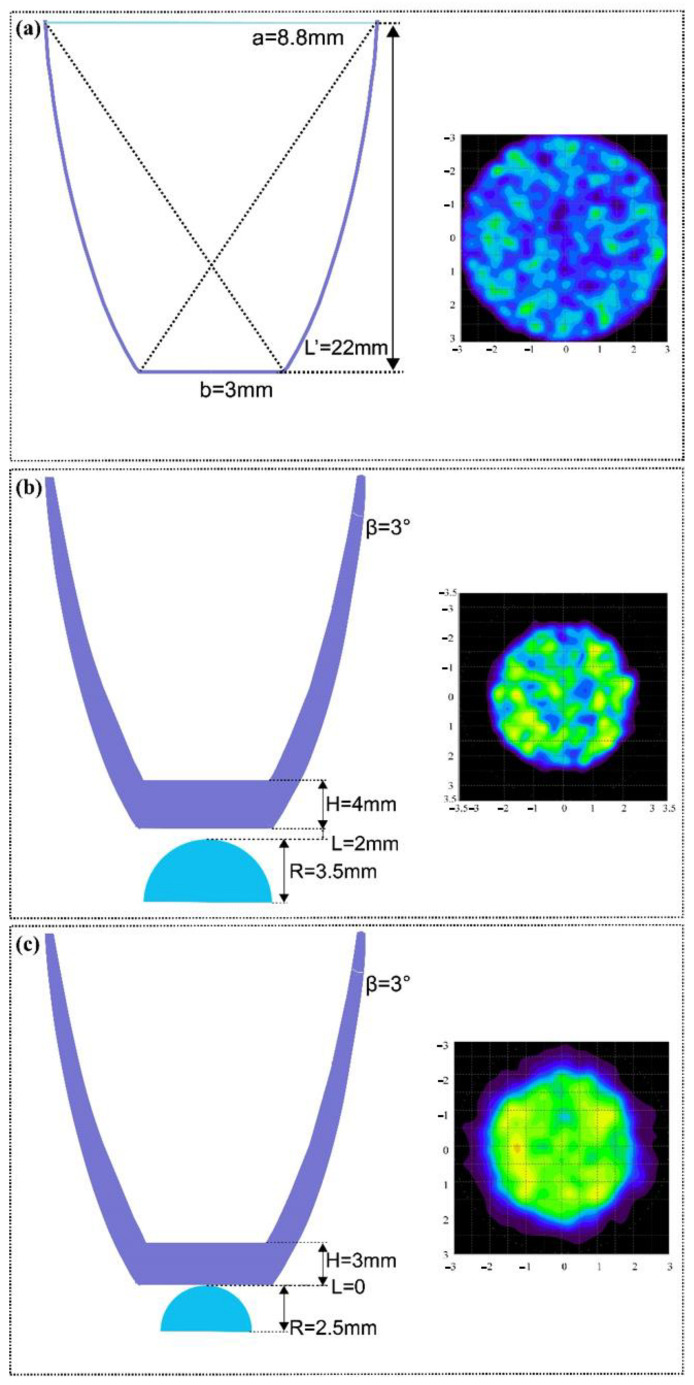
The sectional view of the following optical receiver structures: (**a**) CPC; (**b**) initial structure; (**c**) optimally designed structure.

**Figure 12:**
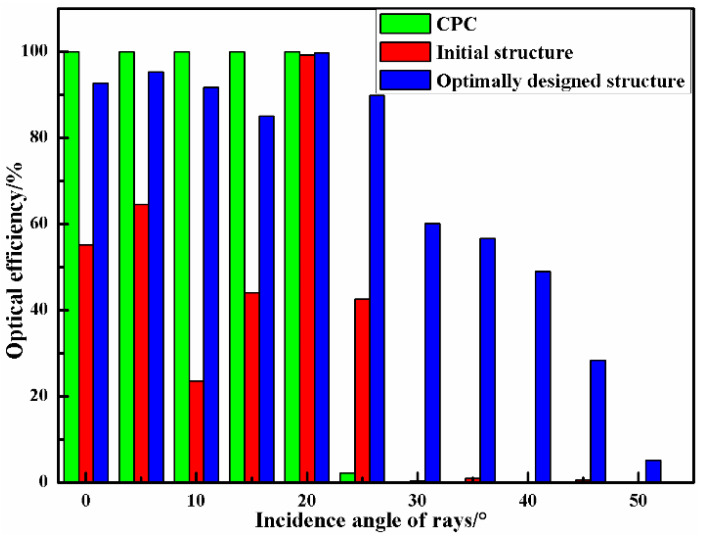
Optical efficiency comparison of the CPC, initial structure, and optimally designed structure for the optical receiver.

**Figure 13:**
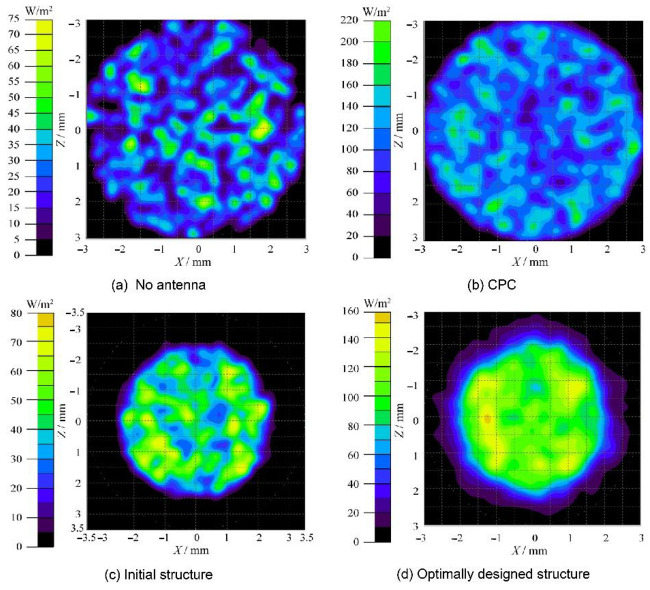
Diagram of the spot areas: no receiver (**a**); CPC (**b**); initial structure (**c**); optimally designed structure (**d**).

As presented in Table 9, the optimum parameter combination is A1-B1-C1-D2. The confirmation operation of the initial structure and the optimized design structure of the optical receiver are compared. Figure 11 presents the sectional view of the CPC, the initial structure, and the optimally designed structure. The S/N ratios of the optimally designed optical receiver have increases of approximately 13.601, 11.097, and 0.373 corresponding to the G, P, and SNR, respectively. Figure 12 presents the optical efficiency data for different optical receiving devices in this study, which indicates that the optimally designed optical receiver has a significantly larger FOV. The optical efficiency remains at a fairly high level even though the incident angle is up to 45°. As the incidence angles are 35°, 40°, and 45°; the optical efficiencies of the initial structure are 0.92%, 0.12%, and 0.58%; and the optical efficiencies of the optimally designed structure are 56.70%, 48.97%, and 28.35%, respectively. The FOV of the optimally designed receiver can increase by nearly 40° over the initial structure. In addition, the spot areas of different conditions are shown in Figure 13. The energy received by the CPC is scattered in the surrounding area, and the uniformity of the spot energy distribution is poor. The initial structure has a smaller spot than the CPC, but the G is just 2.21. In comparison, the spot radius of the optimally designed optical receiver is just 2 mm, and the G is 10.57, which is 4.78 times the initial structure. Therefore, the above analysis results illustrate that the proposed optimization method can effectively obtain the optimum structure parameter combination of the optical receiver.

## 4. Channel Simulation and Analysis with the Optical Receiver

The software of Tracepro and Matlab were utilized to perform the channel modeling and analysis of the indoor VLC system. The LED arrays were set corresponding to the principle of the minimum mean square error of illumination [20], and the center coordinates of the four LED array units are A (0.815, 0.815), B (4.185, 0.815), C (4.185, 4.185) and D (0.815, 4.185), respectively.

### 4.1. Analysis of the Optical Received Power

In the VLC system, LED lights have the function of illuminance and communication simultaneously; the gain of the channel is directly related to the impulse response, and can be represented as follows [25]:

(16) $$H\left(0\right)\;=\;\int_{-\infty }^{\infty }h\left(t\right)dt$$

In an optical link, the channel DC gain can be expressed following Equation (17) [35]:

(17) $$H_{LOS}\left(0\right)\;=\;\left\{\begin{array}{l} \overset{N}{\underset{i=1}{\sum }}\frac{\left(m\;+\;1\right)A_{R}}{2\pi L_{i}^{2}}{cos}^{m}(\phi_{i})Ts\left(\theta_{i}\right)g(\theta_{i})cos(\theta_{i})\\ 0 \end{array}\right.\begin{array}{c} ,\\ , \end{array}\begin{array}{c} \left(0\;\leq \;\theta_{i}\;\leq \;\theta_{c}\right)\\ \left(\theta_{i}\;≻\;\theta_{c}\right) \end{array}$$

where *i* is the *i*-th LED, *T_S_*(*θ_i_*) is the filter gain, *ϕ_i_* is the angle of irradiance, *g*(*θ_i_*) is the DC gain of the concentrator, *A_R_* is the physical area of the detector in a PD, and *θ_c_* is the HFOV of the optical receiving end. The optical concentrator can be expressed as follows [35]:

(18) $$g\left(\theta_{i}\right)\;=\;\left\{\begin{array}{c} \frac{n^{2}}{{sin}^{2}(\theta_{c})}\\ 0 \end{array}\right.\begin{array}{c} ,\\ , \end{array}\begin{array}{c} \left(0\;\leq \;\theta i\;\leq \;\theta c\right)\\ \left(\theta i\;≻\;\theta c\right) \end{array}$$

where *n* represents the refractive index.

The received optical power can be expressed as follows:

(19) $$P_{R}(LOS)\;=\;P_{S}H_{LOS}\left(0\right)$$

where *P_s_* represents the electric power of a single LED.

The direct line-of-sight (LOS) occupies more than 95% of the optical power, and a wall reflection accounts for probably 3.37%, while the secondary wall reflection only accounts for 1.27% of the non-direct line-of-sight (NLOS) based on the study of Toshihiko [35]. Therefore, the secondary wall reflection is negligible, and the gain of the channel can be represented by following Equation (20):

(20) $$dH_{NLOS}\left(0\right)\;=\;\left\{\begin{array}{c} \overset{N}{\underset{i=1}{\sum }}\frac{\left(m\;+\;1\right)A_{R}}{2\pi D_{1,i}^{2}D_{2}^{2}}\rho {cos}^{m}(\phi_{i})cos(\gamma_{1},i)cos(\gamma_{2})T_{S}\left(\theta \right)g(\theta )cos(\theta )dA_{wall}\\ 0 \end{array}\right.\begin{array}{c} ,\\ , \end{array}\begin{array}{c} \left(0\;\leq \;\theta \;\leq \;\theta i\right)\\ \left(\theta \;≻\;\theta i\right) \end{array}$$

where *D_1,i_* represents the distance between the *i*-th LED and a certain point on the wall, *γ_1,i_* is the angle between the light incident on the wall of the *i*-th LED emitting unit, ρ is the wall reflectivity, and d*A_wall_* is the reflective area of a small region.

The receiving power of the indoor VLC system can be represented as follows:

(21) $$P_{R\left(NLOS\right)}\;=\;\int P_{S}dH_{\left(NLOS\right)}\left(0\right)$$

Thus, the total optical receiving power of the optical receiving system can be expressed as follows:

(22) $$P\;=\;P_{S}H_{\left(LOS\right)}\left(0\right)\;+\;\int P_{S}dH_{\left(NLOS\right)}\left(0\right)$$

**Figure 14:**
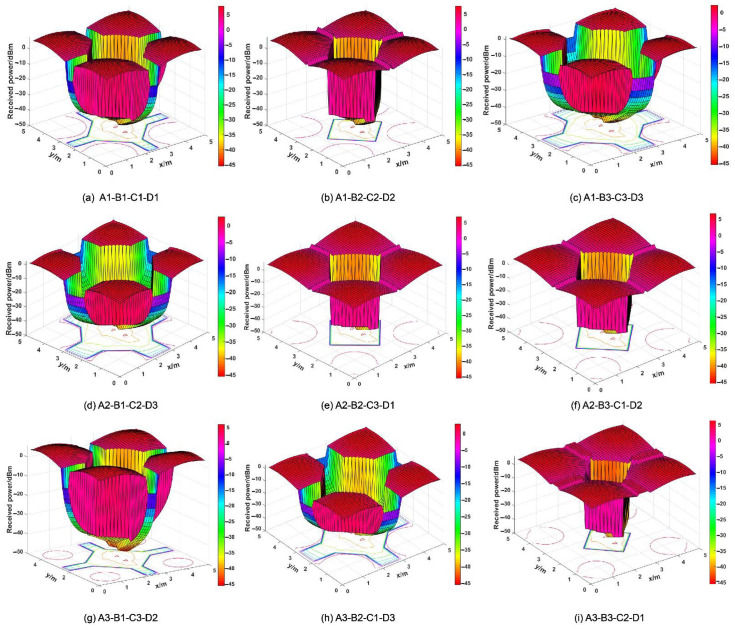
Diagram of the received power distribution for different parameter combinations.

**Figure 15:**
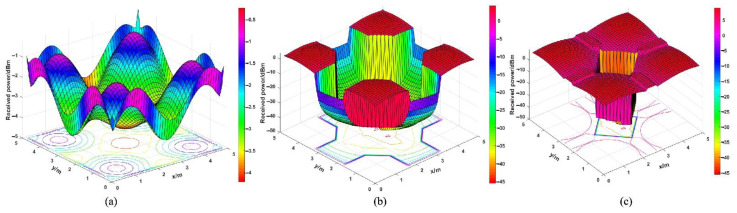
Diagram of the received power distribution: no antenna (**a**), the initial structure (**b**), the optimally designed structure (**c**).

Figure 14 shows the received power distribution for different parameter combinations. It can be seen that different structure settings may have large differences regarding the optical received power. Table 10 presents the maximum, mean, and minimum values of the optical received power under different conditions. Figure 15a shows the power distribution in the room when the optical receiving antenna is not installed: the minimum value of the received power is −4.2258 dBm, the maximum value is −0.2428 dBm, and the mean value is −2.5052 dBm. The received power presents a received power distribution with lower power at the edge position, with higher values from the near position of the LEDs array in the room. A position close to the LED array has a smaller incidence angle of rays, such that the optical receiving system can receive more rays successfully. Figure 15b,c shows the received power distribution of the CPC and the optimally designed structure, respectively. When the optimal structure is utilized, the minimum value of the received power is 8.9900 dBm, the maximum value is −45.6190 dBm, and the mean value is 6.4635 dBm. Moreover, when compared with the initial structure, the mean received power increases by 458.80%, and the maximum value increases by 308.90%.

As the optimal receiving antenna has a large FOV and a uniform distribution of spot energy, the communication dead zone can be significantly reduced.

### 4.2. Analysis of the SNR Distribution

The noise of the indoor VLC system is mainly additive white Gaussian noise, including shot noise and pre-amplifier noise. The communication quality of the system is mainly affected by shot noise, and the photon generated by the background light is much larger than the signal itself. Therefore, the noise generated by the signal itself can be neglected when the background light is strong [36]. When the background light is weak, the pre-amplifier noise is mainly considered. Shot noise can be expressed as follows:

(23) $$\delta_{shot}^{2}\;=\;2q\gamma (P_{R(signal)}\;+\;P_{R\left(ISI\right)})B\;+\;2qI_{bg}I_{2}B$$

where *I_bg_* is the background current, *I_2_* is noise bandwidth factors, *B* is the equivalent noise bandwidth, and *q* is the electronic charge.

The thermal noise is expressed as follows [36]:

(24) $$\delta_{thermal}^{2}\;=\;\frac{8\pi kT_{k}\eta A_{R}I_{2}B^{2}}{G}\;+\;16\pi^{2}kT_{k}\Gamma \eta^{2}A_{R}^{2}I_{3}B^{3}/g_{m}$$

where *k* is Boltzmann’s constant, *g_m_* is the FET transconductance, *G* is the open-loop voltage gain, *Γ* is the FET channel noise factor, and *T_K_* is the absolute temperature.

Thus, the total noise of the system can be obtained as follows:

(25) $$N_{total}\;=\;\delta_{shot}^{2}\;+\;\delta_{thermal}^{2}\;+\;\gamma^{2}P_{R(ISI)}$$

Furthermore, the *SNR* expression of the indoor VLC system can be represented as:

(26) $$SNR\;=\;\frac{\gamma^{2}P_{R\left(signal\right)}^{2}}{\delta_{shot}^{2}\;+\;\delta_{thermal}^{2}\;+\;\gamma^{2}P_{R(ISI)}}$$

The simulation was conducted by using Matlab to compute the *SNR* distribution in the room according to Equation (26), and the specific values of the parameters are listed in Table 11, respectively.

**Figure 16:**
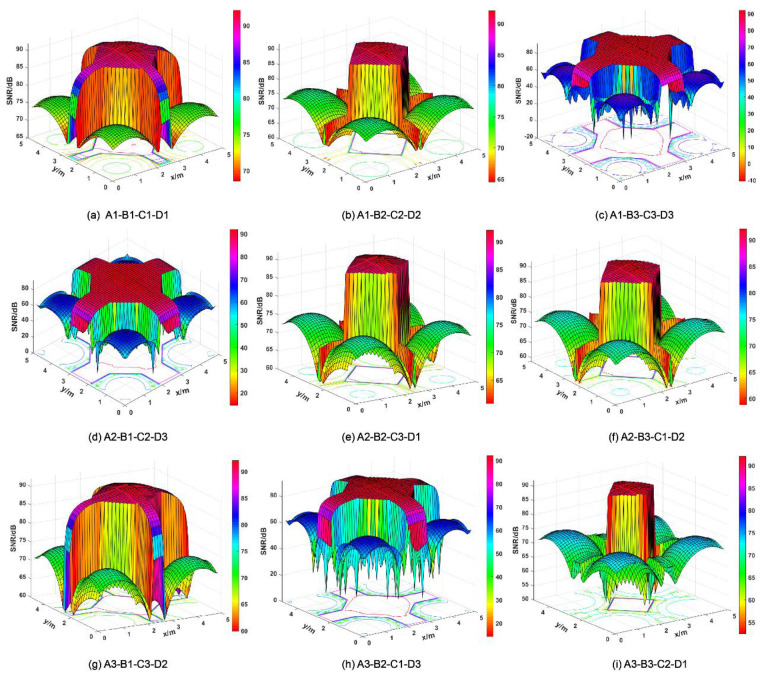
Diagram of the SNR distribution for different parameter combinations.

**Figure 17:**
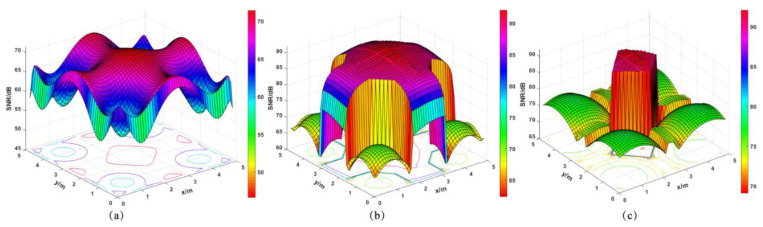
Diagram of the SNR distribution: no antenna (**a**), the initial structure (**b**), the optimally designed structure (**c**).

The SNR in the three cases of an unmounted optical antenna, a mounted CPC, a mounted initial structure, and an optimally designed structure is obtained by calculation, as shown in Table 12. Figure 16 presents the SNR distribution for different parameter combinations. Figure 17a shows the SNR distribution in the room when the optical receiving antenna is not installed; the minimum value of the received power is −4.2258 dBm, the maximum value is −0.2428 dBm, and the mean value is −2.5052 dBm. Figure 17b,c shows the SNR distribution of the CPC and the optimally designed optical receiver, respectively. The *SNR* is calculated by Equation (22), and the results show that the maximum value of SNR is 92.1048 dB, the minimum value is 69.0869 dB, and the mean value is 89.8874 dB. Compared with the case of receiving rays directly without any antenna, the maximum SNR increases by 20.53%, the minimum *SNR* increases by 48.16%, and the mean SNR increases by 33.49%. Moreover, when compared with the initial structure, the minimum SNR increases by 377.38% and the mean SNR increases by 4.39%. In the OOK modulated VLC systems, the minimum required value of *SNR* is 13.6 dB [37]. As can be seen from Figure 17c, the SNR distribution in the room meets the communication requirements. The optimally designed structure with Taguchi and fuzzy logic methods can significantly ensure that more rays can be collected, and can improve the SNR performance.

Finally, the comparison of the optical receiver characteristics using the traditional CPC, the lens-walled structure, and the proposed system were investigated, as presented in Table 13. The relative parameters values of the proposed optical receiver are an FOV of 90°, an optical gain of 10.57, a spot size of 2 mm, a received power of 6.4635 dBm, and an SNR of 89.8874 dB, while those of the traditional CPC are 40°, 3.85, 3 mm, 0.3267 dBm and 68.5486 dB, and those of the lens-walled structure are 80°, 8.62, 4 mm, 5.9484 dBm and 82.8563 dB, respectively. This further proves that the proposed composite optical receiver based on the Taguchi and fuzzy logic methods can improve the optical characteristics and eusure better communication quality.

## 5. Conclusions

In this study, we investigated a composite optical receiver for indoor VLC systems, which can significantly extend the FOV and ensure better channel performance. Furthermore, the Taguchi and fuzzy logic combination method was proposed to improve multiple performance characteristics effectively in the optical receiver, and to meet the communication requirements in VLC. The effects of different β, H, L, and R on the improvement of the optical efficiency and the channel performance of an indoor VLC system using optical receivers were investigated. Meanwhile, the optical and channel performances of the CPC, the initial structure, and the optimized structure of the optical receiver were compared. The simulation results indicate that the optimum composite optical receiver is at the structure parameter combination of A1 (β = 3°), B1 (H = 3.0 mm), C1 (L = 0), and D2 (R = 3.0 mm), which can achieve a G of 10.57, an HFOV of 45°, a P of 6.4635 dBm, an SNR of 89.8874 dB, and a spot size of 2 mm. The FOV increased by 80.00% in comparison with the initial structure, and the mean received power rose by 458.80%. Furthermore, the SNR rises by 33.49% in comparison with directly receiving lights. Therefore, the optical receiver optimally designed by Taguchi and the fuzzy logic methods can significantly meet the requirements of an indoor VLC system.

## Figures and Tables

**Table 1:** Parameters of CPC.

Outlet Radius b/mm	Inlet Radius a/mm	HFOV/°	Length L’/mm	Focal Length f/mm
3	8.8	20	22	4

**Table 2:** Control factors and level settings (L9).

Control Factors		Levels		
		1	2	3
A	Rotation degree β (°)	3.0	4.0	5.0
B	Bottom wall’s height H (mm)	3.0	3.5	4.0
C	Spacing distance L (mm)	0.0	1.0	2.0
D	Hemispherical radius R (mm)	2.5	3.0	3.5

**Table 3:** Taguchi experiments and the performance evaluation.

Exp. No.	Control Factors				Performance Evaluation of Different Characteristics					
	A	B	C	D	G	S/N of G	P /dBm	S/N of P	SNR/ dB	S/N of SNR
1	3.0	3.0	0.0	2.5	8.08	18.153	4.5754	13.209	84.8514	38.573
2	3.0	3.5	1.0	3.0	7.64	17.661	4.7475	13.529	83.1898	38.401
3	3.0	4.0	2.0	3.5	2.21	6.880	−1.8014	5.112	86.1109	38.701
4	4.0	3.0	1.0	3.5	2.42	7.670	−1.4072	2.967	86.1146	38.702
5	4.0	3.5	2.0	2.5	6.54	16.307	4.0724	12.197	83.0983	38.392
6	4.0	4.0	0.0	3.0	6.17	15.812	3.8195	11.640	83.0668	38.389
7	5.0	3.0	2.0	3.0	5.16	14.245	2.6278	8.392	84.6981	38.557
8	5.0	3.5	0.0	3.5	2.56	8.152	−0.8355	−1.561	85.5931	38.649
9	5.0	4.0	1.0	2.5	5.61	14.978	3.7123	11.393	81.2661	38.198

**Table 4:** Normalization of the different quality characteristics.

Exp. No.	Control Factors				Quality Characteristics		
	A	B	C	D	G	P	SNR
1	3.0	3.0	0.0	2.5	1	0.979	0.744
2	3.0	3.5	1.0	3.0	0.925	1	0.404
3	3.0	4.0	2.0	3.5	0	0.442	0.998
4	4.0	3.0	1.0	3.5	0.035	0.300	1
5	4.0	3.5	2.0	2.5	0.737	0.912	0.385
6	4.0	4.0	0.0	3.0	0.675	0.875	0.378
7	5.0	3.0	2.0	3.0	0.502	0.659	0.713
8	5.0	3.5	0.0	3.5	0.059	0	0.894
9	5.0	4.0	1.0	2.5	0.579	0.858	0

**Table 5:** Fuzzy rules for the input and output of the receiver structures.

Test No.	PC1	PC2	PC3	MPCI
1	S	S	S	VS
2	S	M	M	NS
3	S	L	L	S
4	M	S	M	ND
5	M	M	L	M
6	M	L	S	HD
7	L	S	L	L
8	L	M	S	HL
9	L	L	M	VL

**Table 6:** Results of the MPCI for the Taguchi experiments.

Exp. No.	PC1	PC2	PC3	MPCI
1	1.000	0.979	0.744	0.914
2	0.925	1.000	0.404	0.938
3	0.000	0.442	0.998	0.517
4	0.035	0.300	1.000	0.525
5	0.737	0.912	0.385	0.539
6	0.675	0.875	0.378	0.821
7	0.502	0.659	0.713	0.597
8	0.059	0.000	0.894	0.511
9	0.579	0.858	0.000	0.658

**Table 7:** Response of the MPCI tables.

	A	B	C	D
Level 1	0.790	0.679	0.749	0.704
Level 2	0.628	0.663	0.707	0.785
Level 3	0.589	0.665	0.551	0.518
max-min	0.201	0.016	0.198	0.268
Rank	2	4	3	1

**Table 8:** Variance analysis on the MPCI results.

Factors	Sum of Squares	Degree of Freedom	Mean of Squares	Contributions (%)
A	8.837	2	4.419	29.46%
B	0.703	2	0.352	2.34%
C	8.691	2	4.346	28.97%
D	11.768	2	5.884	39.23%

**Table 9:** Comparison between the initial structure and the optimally designed structure.

Characteristics	Initial Structure A1-B3-C3-D3	Optimally Designed Structure A1-B1-C1-D2	Gain
G	6.880	20.481	13.601
P	5.112	16.209	11.097
SNR	38.701	39.074	0.373

**Table 10:** Receiving power comparison of different antenna structures.

Systems	P_Rmax_ [dBm]	P_Rmin_ [dBm]	P_Rave_ [dBm]
No-antenna	−0.2428	−4.2258	−2.5052
CPC	4.1929	−45.6190	0.3267
Initial structure	2.1986	−45.6190	−1.8014
Optimally designed structure	8.9900	−45.6190	6.4635

**Table 11:** The simulation and design parameters of the indoor VLC system.

Parameter Types	Parameter Values
FET channel noise factor *Γ*	1.5
Background current *I_bg_*	5.1 mA
Fixed capacitance of photodetector per unit area *η*	112 pF/cm^2^
Noise bandwidth factors *I_2_*	0.562
FET transconductance *g_m_*	30 Ma
Noise bandwidth factors *I_3_* [35]	0.0868
Open-loop voltage gain *G*	10.0
Equivalent noise bandwidth *B*	100 Mbit/s
Absolute temperature *T_K_*	298 K

**Table 12:** Signal-to-noise ratio comparison of different antenna structures.

Systems	SNR_Rmax_ [dB]	SNR_Rmin_ [dB]	SNR_Rave_ [dB]
No-antenna	71.4401	46.6286	67.3383
CPC	92.1048	62.3373	86.7153
Initial structure	92.1048	14.4722	86.1109
Optimally designed structure	92.1048	69.0869	89.8874

**Table 13:** Comparison results of three different design methods.

Optical Receiver	FOV [°]	Optical Gain	Spot Size [mm]	Received Power [dBm]	SNR [dB]
CPC	40	3.85	3	0.3267	68.5486
Lens-walled structure	80	8.62	4	5.9484	82.8563
Proposed optical receiver	90	10.57	2	6.4635	89.8874

## Data Availability

The data presented in this study are available in this published article.
